# Acute airway inflammation following controlled biodiesel exhaust exposure in healthy subjects

**DOI:** 10.1186/s12989-024-00614-5

**Published:** 2024-12-05

**Authors:** Thomas Sandström, Jenny A. Bosson, Ala Muala, Mikael Kabéle, Jamshid Pourazar, Christoffer Boman, Gregory Rankin, Ian S. Mudway, Anders Blomberg, Maria Friberg

**Affiliations:** 1 Department of Public Health and Clinical Medicine, Umeå University, SE-901 87, Umeå, Sweden; 2 Thermochemical Energy Conversion Laboratory, Department of Applied Physics and Electronics, Umeå University, Umeå, Sweden; 3 Swedish Defence Research Agency, Umeå, Sweden; 4 NIHR Health Protection Research Unit in Environmental Exposures and Health, MRC-PHE Centre for Environment and Health, Imperial College London, London, UK

**Keywords:** Air pollution, Lung, Chamber exposure, Biodiesel, Renewable fuel, Bronchoscopy, Bronchial biopsy

## Abstract

**Background:**

Exposure to standard petrodiesel exhaust is linked to adverse health effects. Moreover, there is a mounting request to replace fossil-based fuels with renewable and sustainable alternatives and, therefore, rapeseed methyl ester (RME) and other biofuels have been introduced. However, recent toxicological research has indicated that biodiesel exhaust may also induce adverse health-related events.

**Aim:**

To determine whether exposure to 100% RME biodiesel (BD100) exhaust would cause an acute airway neutrophilic recruitment in humans.

**Methods:**

Fourteen healthy subjects underwent exposure to diluted BD100 exhaust and filtered air for 1-h, in a blinded, random fashion. Bronchoscopy with endobronchial mucosal biopsies, bronchial wash (BW) and bronchoalveolar lavage (BAL) was performed six hours after exposure. Differential cell counts and inflammatory markers were determined in the supernatant and biopsies were stained immunohistochemically.

**Results:**

Compared with filtered air, BD100 exhaust exposure increased bronchial mucosal endothelial P-selectin adhesion molecule expression, as well as neutrophil, mast cell and CD68 + macrophage numbers. An increased influx of neutrophils and machrophages was also seen in BW.

**Conclusion:**

Exposure to biodiesel exhaust was associated with an acute airway inflammation that appeared similar to preceding petrodiesel exposure studies. The present findings, together with the recently reported adverse cardiovascular effects after similar biodiesel exposure, indicate that biodiesel is not free of toxicity and may affect human health.

**Supplementary Information:**

The online version contains supplementary material available at 10.1186/s12989-024-00614-5.

## Introduction

Air pollution is a key global predicament, contributing to the deterioration and worsening of respiratory and cardiovascular diseases, as well as causing a wide range of complementary organ manifestations and metabolic changes [1–3]. WHO has estimated 400,000 deaths per year in Europe, and over 7 million annually worldwide, to be associated with air pollution [4].

As climate change and dependence on diminishing fossil fuel supplies have gained increasing attention, renewable biofuels, such as biodiesel, have been suggested as beneficial and more sustainable alternatives. It may also be anticipated that exhaust from renewable “green fuels” would be less harmful to human health than exhaust from petroleum-based diesel fuels [5]. The latter, often referred to as petrodiesel, when used in vehicle engines, produces diesel exhaust particulates that have been indicated as an important contributor to the adverse health effects of air pollution [1, 5, 6].

Adverse effects of petrodiesel exhaust particles have been well studied using cell and animal models [7, 8]. The use of controlled exposure chamber studies in human subjects, with diesel engines running on petrodiesel fuel has contributed to an increased understanding of the adverse health effects [9–18]. Bronchoscopy sampling in the lungs of human research subjects have shown consistency in terms of neutrophilic airway inflammation shown in bronchial mucosal biopsies and bronchial lavage [16, 19–21]. The underlying pathways for the inflammatory response to petrodiesel in human airways have been well delineated and include phosphorylation of the 1173 site on the epithelial growth factor receptor (EGFR), activation of redox sensitive transcription factor p65 NFkB, MAPkinases and release of neutrophil chemoattractants such as IL-8 and GRO-alpha. Other inflammatory components have also been shown to be involved, such as increase in bronchial epithelial production of the TH2 cytokines IL-4 and IL-13 [22–24]. Asthmatic and healthy subjects have display different responses in airway lymphocytes, eosinophils and IL-10 levels in the airways after petrodiesel, which may be associated with the increased vulnerability of asthmatic airways [25].

The petrodiesel effects in the airways were reconfirmed in a recent investigation repeating the study protocol from the earlier studies, with the same exposure chamber and engine running on standard petrodiesel in healthy human subjects [9]. The bronchial biopsies and lavages showed similar airway inflammatory findings including neutrophilia, but also expanded on the understanding of the role of aryl hydrocarbon receptor (AhR) and CYP1A1 activation in relation to the exhaust exposure. Exposure studies with petrodiesel have also linked detailed cardiovascular effects in human subjects with the multi-peaked timeline for risk of developing myocardial infarctions and stroke after traffic air pollution exposure [12, 13, 26–29]. Reduced ability to dilate blood vessels during stress (vasomotor function), reduced release of anti-thrombotic factors like tissue plasminogen activator (t-PA), increased thrombocyte-monocyte adhesion, increased ex-vivo thrombosis, increased arterial stiffness, ST-T segment depression on ECG, corresponding to mitochondrial disturbance, are examples of cardiovascular mechanisms that contribute to increased risk for adverse health effects.

As regards biodiesel exhaust effects on the cardiovascular system of human subjects, this has only recently been investigated [30]. The study used similar chamber exposures and protocol as earlier for petrodiesel, and investigated the local vascular effects of the commonly used biodiesel fuel rapeseedmethyl ester (RME) as 100% blend vs. filtered air. The next study, included in the same publication in PFT, investigated the effects of petrodiesel exhaust vs. biodiesel head-to-head. The investigation demonstrated biodiesel exposure to cause cardiovascular effects that appeared equal to that of petrodiesel. This was seen despite the exhaust PM emissions during the chamber exposures with RME biodiesel were reduced by half, when the engine was running under the same load and running conditions, as for petrodiesel fuel, in accordance with earlier combustion research [31, 32]. It was therefore of major interest to also determine whether exposure to biodiesel exhaust would cause similar or different airway effects in human subjects, in comparison to filtered air.

The main hypothesis was that exposure to exhaust from a diesel engine running on 100% RME biodiesel (BD100) would cause an acute airway inflammatory response in terms of airway neutrophilia, reflected in the bronchial mucosa and bronchial wash (BW), compared to filtered air exposure in healthy human subjects. These endpoints were based on the previous studies that have reported airway outcomes of petrodiesel exhaust exposure [9, 16–18, 21–24, 33–35].

## RESULTS

### Exposures

The BD100 exhaust exposures were performed at a filter-based PM_10_ (particulate matter less than 10 μm in diameter) mass concentration of 166 ± 31 μg/m^3^ (mean + SD) (corresponding to 178 ± 17 μg/m^3^ measured with a TEOM), NO_2_ 0.57 ± 0.13 ppm, NO_x_ 7.00 ± 0.76 ppm and a total gaseous hydrocarbon (THC) concentration of 0.76 ± 1.15 ppm. A detailed description of exposure characteristics has been reported in the companion paper by Unosson et al., which was carried out with similar exposure conditions [30].

### Biopsies

**Fig. 1:**
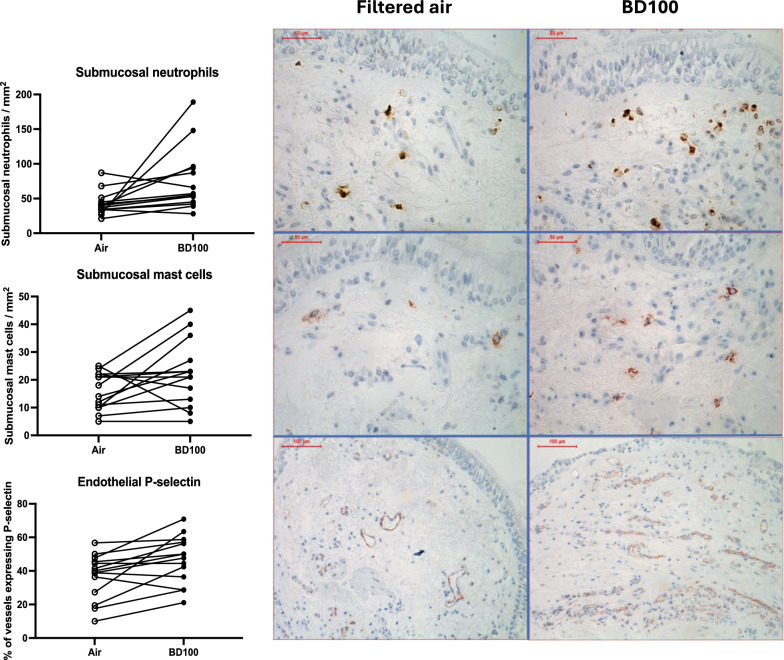
Immunohistochemistry of GMA embedded bronchial mucosal biopsies. BD100 exhaust exposure caused increased cell influx and adhesion molecule expression, compared to filtered air. Upper panel shows neutrophil elastase staining, upper left photo post air exposure and upper right post BD100 exhaust exposure. Middle panel shows mast cell tryptase staining after air (middle left) and BD100 exhaust exposure (middle right). Lower panel shows p-selectin adhesion molecule endothelial staining after air (lower left) and BD100 exhaust exposure (lower right). Photos in the upper and middle panels (cell stainings) have been taken at × 40 magnification and the scale bar represents 50 μm. Photos in the lower panel (adhesion molecule expression) have been taken at × 20 magnification and the scale bar represents 100 μm

Immunohistochemical analysis of bronchial biopsies demonstrated that BD100 exhaust exposure caused a significant increase in the vascular endothelial expression of the adhesion molecule P-selectin (*p* = 0.007), together with a non-significant trend towards an increase in the ICAM-1 expression (*p* = 0.064) compared to filtered air (Table 1, Fig. 1).

**Table 1:** Inflammatory markers in bronchial mucosal biopsies

	Neutrophils *epithelium*	Neutrophils *submucosa*	Mast cells* submucosa*	Eosinophils* submucosa*	CD68 *submucosa*	CD4* submucosa*	CD8* submucosa*	CD4/CD8* submucosa*	P-selectin* submucosa*	ICAM-1* submucosa*
Filtered Air	0.00	38.2	16.1	0.00	0.00	27.2	24.2	1.17	39.4	40.4
	0.00–0.84	33.0–47.0	10.3–22.2	0.00–0.46	0.00–2.25	20.0–42.2	15.4–41.7	0.75–1.59	25.3–46.0	28.2–50.4
BD100	0.89	56.2	21.7	0.00	1.86	29.5	42.1	0.74	48.8	48.8
	0.00–4.07	44.2–94.0	12.5–29.7	0.00–7.14	0.00–3.50	20.6–45.2	23.0–58.0	0.62–0.90	34.4–57.5	41.0–53.9
*p*-value*	0.038	0.009	0.030	0.069	0.021	0.470	0.096	0.030	0.007	0.064

Cell counts in bronchial epithelium (cells/mm epithelium) and submucosa (cells/mm^2^) as well as adhesion molecule expression in per cent of the pan-endothelial EN4 marker in the submucosa of bronchial mucosal biopsies. Data are given as median with interquartile range. *Wilcoxon signed rank test. Data are given for filtered air and BD100 exhaust exposure

**Fig. 2:**
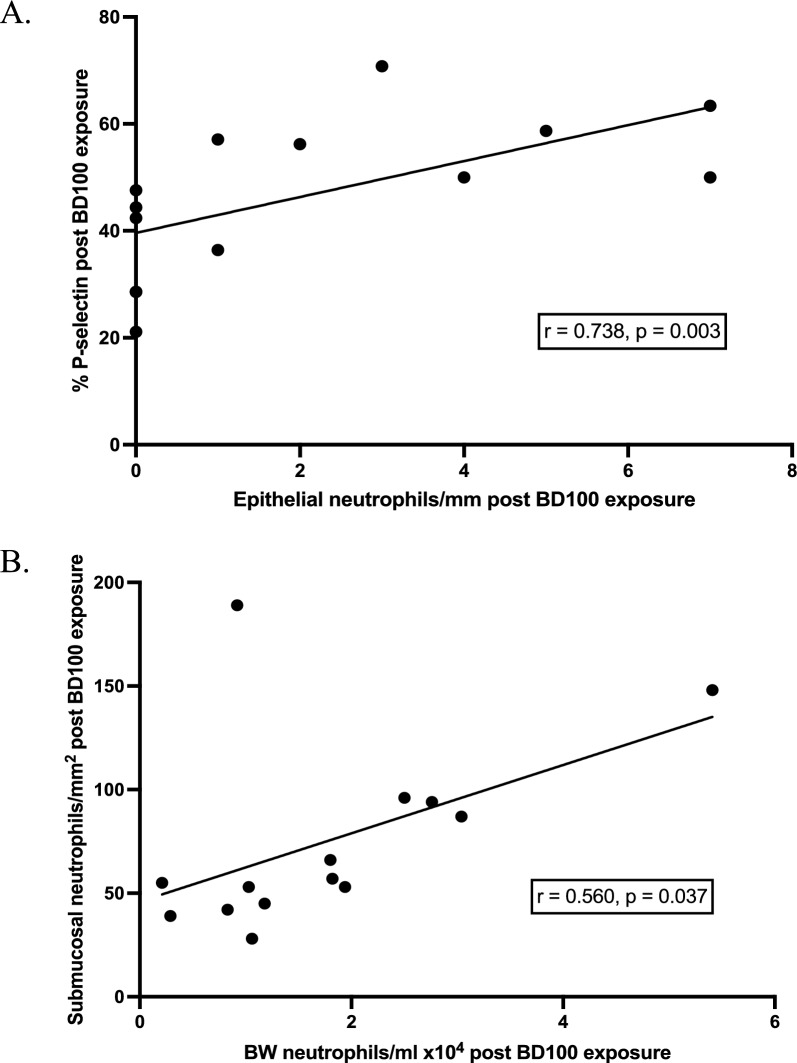
Associations between inflammatory markers in endobronchial mucosal biopsies and bronchial wash. Spearman’s rank correlations. A *p*-value < 0.05 was considered significant (N = 14). **A** Association between endothelial P-selectin expression and epithelial neutrophil numbers in endobronchial mucosal biopsies after BD100 exhaust exposure (r = 0.74, *p* = 0.003). **B** Association between neutrophil numbers in the bronchial submucosa and bronchial wash (BW) after BD100 exhaust exposure (r = 0.560, *p* = 0.037)

The adhesion molecule responses were accompanied by an acute neutrophil influx into the bronchial submucosa (*p* = 0.009) and bronchial epithelium (*p* = 0.038) after BD100 exhaust exposure. There was a significant association between endothelial P-selectin and epithelial neutrophils after BD100 exhaust exposure (r = 0.74, *p* = 0.003, Fig. 2). Other types of inflammatory cells were few in the bronchial epithelium, with no significant difference between exposures (data not shown).

In the bronchial mucosa, there were also significant influxes of mast cells and CD68 + macrophages (*p* = 0.030 and *p* = 0.021 respectively) after exposure to BD100 exhaust vs. filtered air (Table 1). While CD4 and CD8 cell counts were not significantly changed by exposures, the CD4 + /CD8 + lymphocyte ratio decreased after BD100 exhaust exposure (*p* = 0.030) when compared to air. This was mainly driven by a non-significant increase in CD8 + cells.

### BW and BAL

A statistically significant increase in BW neutrophil numbers was observed after BD100 exhaust exposure compared to filtered air (*p* = 0.009, Table 2), which also had a moderate correlation with submucosal neutrophil numbers (r = 0.560, *p* = 0.037, Fig. 2).

**Table 2:** Cell counts in bronchial wash

	Neutrophils	Macrophages	Lymphocytes	Eosinophils	Mast Cells
Filtered Air	0.85	5.16	0.24	0.00	0.013
	0.56–1.64	3.35–7.91	0.12–0.53	0.00–0.004	0.007–0.022
BD100	1.49	8.97	0.37	0.03	0.010
	0.90–2.56	3.08–12.93	0.07–0.74	0.00–0.02	0.003–0.022
*p*-value*	0.009	0.026	0.331	0.401	0.552

Cell counts in BW. Data are given as cells × 10^4^/ml expressed as median with interquartile range. *Wilcoxon signed rank test**. **Data are given for filtered air and BD100 exhaust exposure

Macrophages in BW were also significantly increased after biodiesel vs. air (*p* = 0.026), with other cell types unaffected by exposures. BAL cell numbers did not differ between exposures (data not shown).

The levels of neutrophil myeoloperoxidase (MPO), and the metalloproteases MMP-9 and MMP-12 and their inhibitors TIMP1 and TIMP2, did not differ significantly in either BW or BAL between the two exposures, at the investigated 6-h time point (Table 3).

**Table 3:** Soluble components in bronchial wash and bronchoalveolar lavage

	MPO ng/ml		MMP-9 ng/ml		MMP-12 pg/ml		TIMP1 ng/ml		TIMP2 pg/ml	
	*BW*	*BAL*	*BW*	*BAL*	*BW*	*BAL*	*BW*	*BAL*	*BW*	*BAL*
Filtered Air	64.8	1.40	2.55	0.60	628	634	6.35	0.50	745	559
	25.0–131.5	0.92–2.32	1.55–4.12	0.60–0.62	525–768	468–762	3.97–9.80	0.50–0.80	519–989	467–713
BD100	104.3	2.10	4.05	0.65	729	588	8.00	0.60	808	574
	48.6–171.7	1.10–2.62	2.25–7.80	0.60–0.92	614–844	517–676	4.82–11.92	0.50–0.95	619–969	463–812
p-value*	0.43	0.39	0.22	0.19	0.24	0.83	0.15	0.14	0.40	0.70

Myeloperoxidase (MPO) and metalloproteases (MMP) 9 and 12, together with metalloprotease inhibitors (TIMP) 1 and 2 in BW and BAL fluid after exposure to filtered air and BD100 exhaust exposure. Data are given as median with interquartile range. *Wilcoxon signed rank test

BD100 exposure vs. filtered air showed significantly increased levels of the lipid mediators prostaglandin E_2_ (PGE_2_) (*p* < 0.001), 12,13-dihydroxyoctadecenoic acid (12,13-DiHOME) (*p *< 0.01) and 13-hydroxyoctadecadienoic acid (13-HODE) (*p* < 0.01), as previously reported [36]. In BW, 9-hydroxyoctadecadienoic acid (9-HODE) was increased after BD100 vs. air (*p* < 0.05).

### Spirometry

Dynamic spirometry did not demonstrate any significant lung function differences between the two exposures (see Additional file 1), in similarity with the recent companion biodiesel study using the same exposure protocol, but focusing on reporting cardiovascular endpoints [30].

## Discussion

Controlled chamber exposure of healthy human subjects to BD100 exhaust triggered airway inflammatory events including airway neutrophilia, in addition to the cardiovascular consequences recently reported in a companion study, employing a similar exposure [30]. These respiratory and cardiovascular effects were seen, despite the fact that the use of the RME-based biodiesel fuel resulted in roughly half of the PM mass concentration in the chamber, as compared to petrodiesel. As illustrated in the companion study by Unosson et al. 2021 [30], the BD100 exhaust contained higher concentration of smaller nanoparticles, with shift in size distribution from mono-modal (peak at 100–140 nm) for the petrodiesel, to bi-modal (peaks at 30–40 nm and 80–90 nm) for the BD100 exhaust. This was based on the engine running at the same load and speed, according to a standardised urban traffic running cycle, during all diesel exposures.

The preceding petrodiesel exhaust exposure studies in humans have consistently shown a neutrophilic response in the bronchial mucosa, epithelium and bronchial wash, which was also confirmed in the present study after exposure to BD100 exhaust (see Table 4 for comparison). The upstream pathways indicated for petrodiesel-induced neutrophil recruitment, including EGFR phosphorylation, activation of AhR, CYP1A1, p65 NFкB, and MAPkinase, as well neutrophil chemoattactants, may also be involved after biodiesel exhaust exposure [9, 23, 24, 35]. The increased expression of vascular adhesion molecules, providing rolling and firm adhesion for the recruitment of neutrophils and monocytes from the blood stream to the bronchial mucosa after BD100 exhaust exposure, was also in line with previous petrodiesel exhaust exposure studies [9, 16, 17].

**Table 4:** Comparison of airway inflammatory effects between biodiesel and petrodiesel exhaust exposure

	BD100 versus air *Present study*	Petrodiesel versus air *Salvi *et al*. AmJRCCM 1999* (8)	Petrodiesel versus air *Friberg *et al*. PFT 2023* (15)
*Bronchial biopsy*			
P-selectin subm	+ +	ns	ND
ICAM-1 subm	( +)	+ +	ND
Neutrophil subm	+ +	+ +	+ +
Neutrophil epi	+	+ +	ND
Mast cells subm	+	+	+
CD68 + Macrophage subm	+	ND	ND
Lymphocytes subm	ns	+	+
*BW*			
Neutrophil	+ +	+ +	+ +
Macrophage	+	ns	ns

+ and +  +  = significant increase at *p* < 0.05 and *p* < 0.01 respectively, ( +) = non-significant trend *p* < 0.07, *ns* non-significant, *ND* no data. Inflammatory effects in the lungs in the current BD100 exhaust exposure study and two previous petrodiesel studies. Similar exposure protocol used with bronchoscopies performed 6 h after controlled diesel exhaust versus filtered air exposures. Findings in bronchial biopsy submucosa (subm) and epithelium (epi), as well as bronchial wash (BW)

A novel finding was the recruitment of CD68 + macrophages into the bronchial mucosa together with an increase in macrophages in BW after BD100 exhaust exposure (Tables 1 and 2). The early appearance of monocytic cells in the bronchial mucosa, appears mediated by upregulated vascular adhesion expression. These cells with phagocytic properties, expressing the scavenger receptor CD68 + , are expected to be of importance for the T-cell interaction after biodiesel particles exposure. At the investigated time point, T-cells were not yet elevated in the bronchial mucosa, but they have been common compatriots in the bronchial inflammation following petrodiesel exposure [9, 16]. When it comes to alveolar macrophages in the peripheral airspaces, sampled by BAL, we did not find any increase at the 6-h time point. This finding is in line with previous petrodiesel research, showing alveolar macrophages to appear *en masse* at a later phase, i.e. 24 h after exposure in human subjects, due to demand for clearance of exhaust particles [18]. While the macrophage recruitment enhances the clearance capacity, we reported petrodiesel exhaust particles to adversely impair the phagocytic capacity of the individual macrophages [18]. This will be addressed in future biodiesel studies, when the later phase outcomes will be investigated.

The influx of macrophages and neutrophils into the airways reflected by the BW, was not accompanied by any increase in the levels of the neutrophil peroxidase MPO or metalloproteases and inhibitors, mainly secreted by neutrophils and macrophages, at the investigated 6-h time point. Forthcoming research will address these components at a later time frame, as indicated by a previous study [37].

In a companion paper [36], it was shown that biodiesel exposure significantly increased BAL-fluid levels of 12, 13-dihydroxyoctadecenoic acid (12,13-DiHOME), a product from linoleic acid, through the CYP pathway and downstream epoxide hydrolase oxidation. This lipokine, released by activated leukocytes, has been associated with the recruitment of neutrophils, lymphocytes and monocytes. BD100 exposure also increased BAL levels of the lipid mediators 13-hydroxyoctadecadienoic acid (13(S)-HODE) and 12,13-dihydroxy-9Z-octadecenoic acid (13-HODE). 13(S)-HODE may be produced during oxidative stress and has been associated with airway epithelial injury, by several pathways including binding to phospholipids in mitochondrial membranes, leading to increased permeability and functional impairment [38]. Interestingly, 13(S)-HODE activates the transient receptor potential cation channel subfamily V 1 (TRPV1), which contributes to autonomic nervous dysfunction and adverse cardiovascular effects, such as impaired vasomotor function, myocardial dysfunction and ST-segment depression on ECG, as previously reported following petrodiesel exposure [12, 15]. The elevated BAL 13(S)-HODE levels may thus suggest similar vascular effects by exposure to BD100 exhaust. Furthermore, we reported a BAL fluid increase in prostaglandin E2 (PGE2) after BD100 exhaust exposure in human subjects, as well as in vitro using a multicell model [39]. PGE2 may have several effects related to petrodiesel- and biodiesel exhaust exposure, including activation of TRPV1, but also impairment of phagocytosis, as previously identified after petrodiesel exposure [18].

So far, few studies have addressed effects of biodiesel exhaust exposure in human subjects. Mehus et al. investigated exposures to diesel exhaust from soy methyl ester (SME) (75% blend in 25% petrodiesel fuel) in 48 subjects in an open heavy load-haul-dump (LHD), during mucking operations underground in a mining environment [37]. PM_10_ concentrations were 336 µg/m^3^ for petrodiesel and 268 µg/m^3^ for SME75 biodiesel during exposure for 200 min, thus a much higher exposure dose than in the present study. Both petrodiesel and SME75 biodiesel reduced FEV_1_ and FVC 6 h after exposure, not considered to be a common finding after experimental petrodiesel studies using lower exposure burdens. The reductions in FEV_1_ and FVC were in the range of 100–200 ml and were marginally less for SME75. Induced sputum analyses showed increases in neutrophils, macrophages, MMP-9 and MPO across the exposures, both for bio- and petrodiesel. As induced sputum collects an airway secretion induced by a strong provocation with hypertonic saline, it does not always reflect the unprovoked state of the lung tissue, as reflected in biopsies and bronchoalveolar lavages. This could together with the probably higher total exposure dose (PM concentration x time) contribute to differences in effects between the findings by Mehus et al. and the present study. Neither can it be excluded that RME and SME may act differentially in the airways, due to different physico-chemical factors. Toxicological effects of exhaust from various biodiesel and petrodiesel fuels have been investigated using animal and cell models, with findings that are largely consistent with the airway and cardiovascular findings from experiemental studies in humans Biodiesel fuels, despite being described as “green”, renewable and CO_2_-neutral, do not necessarily provide less toxic or proinflammatory potential as compared to petrodiesel, when a variety of endpoints are taken into account [39–47].

For comparisons of environmental and health impacts by different fuels, such as biodiesel and petrodiesel, it has been highlighted that it may not be sufficient to rely only on the engine exhaust PM mass equivalence emission factors, e.g. given as g PM per kWh. Since the actual emissions to the ambient air depend on a combination of the PM mass equivalence emission factors per energy (fuel) used and the fuel consumption, an assessment of the emissions per distance for the specific vehicle when using different fuels, could be a more relevant measure to assess real life environmental health impacts [48, 49]. Biodiesel fuels, which consist of fatty acid methyl esters, have higher cetane number than petrodiesel, resulting in faster ignition and combustion. Moreover, an increased ratio of oxygen within the fuel may affect the combustion efficiency, which together with less aromatics and sulphur, contributes to reduced soot formation and particle generation. This has been taken into account when it comes to the design of the present and our preceding studies, in which biodiesel PM concentrations often have been lower compared with corresponding petrodiesel fuel exhaust emissions, given the same engine load and running conditions [30, 36, 37, 41, 42, 49]. In addition, we have shown that the reduction in biodiesel PM mass in the present engine setup was associated with a higher fraction of organic matter, considerable less PAHs but a relatively higher fraction of oxygenated PAHs (oxy-PAHs), as well as higher numbers of small nanoparticles [30, 32].

### Strengths and limitations

Strengths include investigations in human subjects, which provide species validation as compared to other research approaches. Each subject was investigated twice, thereby serving as her or his own control. The random order of exposures was blinded to the subjects and the investigators, but known to the technical staff providing the exposure atmosphere, with codes broken only after completion of statistical analyses. The same engine setup and study protocol was used with comparable running conditions as in a series of earlier studies [17, 30, 50, 51].

Limitations include exposure with 1 h duration, which only gives information regarding acute effects of short-term exposure, whereas long-term repeated exposures are difficult to perform in human subjects under controlled conditions. It is recognised that information of health effect parameters in elderly individuals and subjects with respiratory and cardiovascular diseases is highly relevant from health care perspectives. We have previously undertaken exposure studies in elderly as well as individuals with allergy, asthma, COPD and cardiovascular diseases [12, 33, 34, 52, 53], which would be of importance to study in regard to health effects of exhaust from novel fuels.

It would have been ideal to also include a third exposure arm in terms of petrodiesel exposure. It is however very difficult to perform studies in human subjects that include more than two exposure days and two bronchoscopies, due to discomfort as well as several logistics aspects. We have tried multiple 3–4 repeat bronchoscopies after controlled exposures in human subjects previously, but have found it difficult, unpractical and increasing the risk for subject drop out [21]. The prolongation of such studies with proper wash out periods between bronchoscopies, also adds the problems with the demand of long periods free of virus infections, which affects airway cells, making studies very long with risk of changes of baseline parameters.

The current and previous studies have been exploring diesel engiens running at steady state according to the urban sequence of the European Transient cycle, mimicking urban traffic situations. It does not include a start up phase of the engine, which may be of particular importance during cold spells during winter, when engines may take time to reach more efficient combustion.

This study includes a variety of inflammatory endpoints mainly focused on bronchial tissue and lavages with limitations on what was measured. Complementary panels of markers of inflammation, oxidative burden and other mechanisms are motivated, and remain in focus for forthcoming reports.

Investigations with invasive procedures, such as bronchoscopies in humans, are limited to the number of subjects that can practically be investigated, depending on subject recruitment, staff and financial resources. It is recognised that a larger number of subjects could have provided additional statistical strength. A series of earlier studies with various air pollutants such as nitrogen dioxide, ozone, wood smoke and diesel exhaust have demonstrated the current number of subjects to be sufficient to determine major inflammatory and oxidative events in the lungs. The selected time point for bronchoscopies was based on earlier bronchoscopy studies and is a crucial part of the study design. While it could have been of interest to also determine effects at another time point, that would have demanded a separate investigation with other research subjects, as more than two bronchoscopies in the same individual is cumbersome.

Similarities and differences when it comes to the physical and chemical properties of exhaust from biodiesel and petrodiesel fuels deserve further exploration, in order to determine whether future diesel, or diesel-like fuels, fuels could be designed to avoid the toxicological properties that lead to adverse respiratory and cardiovascular responses.

### Conclusion

Exposure to exhaust from 100% RME fuel used in an existing heavy-duty engine was demonstrated to cause acute inflammatory outcomes reflected in bronchial mucosal biopsies and BW as early as 6 h after exposure. The recruitment of neutrophils and mast cells, together with the increased adhesion molecule expression, was in line with preceding studies employing petrodiesel exhaust exposure.The present bronchoscopy study of lung effects, together with the preceding study of cardiovascular effects in human subjects, indicates that biodiesel exhaust does not appear to be free of toxicity, and may be associated with adverse health effects.

## Material and methods

### Subjects

Fourteen healthy non-smoking subjects, 6 female and 8 male, mean age 25 years (range 19–36 years) completed the study. On a pre-exposure study day, all subjects underwent a bicycle ergometer test including ECG to determine an individual work load in order to achieve a set minute ventilation of 20 L/min/m^2^ body-surface during exposures. Subjects were instructed to refrain from taking vitamins, anti-oxidants and anti-inflammatory agents during the study. Female subjects were using oral anticontraceptives, and pregnancy tests were taken before each exposure. Oral and written informed consent was obtained and the local ethical reviewboard at Umeå University approved the study that was performed in accordance with the Declaration of Helsinki.

### Study design

The research subjects were exposed to diluted exhaust from a diesel engine running on 100% RME biodiesel fuel (BD100) or filtered air in a validated exposure chamber, according to a randomized double-blind crossover protocol [30], with an interval of at least 2 weeks between exposures. Each exposure lasted for 1 h, with rest and exercise alternating at 15-min intervals. A registered nurse or physician monitored the subjects during all exposures. Separately, an engineer monitored and adjusted the exposure concentration in the chamber. During filtered air exposures, the diesel engine was running in the adjacent engine chamber, with the difference that no exhaust was fed into the ventilation shaft leading to the exposure chamber, but the noise from the engine was still present. There is always a smell of diesel exhaust in the chamber, wheather diesel exhaust is fed or not.

### Chamber exposures

As previously described in detail [32], a heavy duty four cylinder 4.0 L Volvo diesel engine TD40 GJE was running under variable speed and load, according to the urban part of the European Transient Cycle, in order to simulate exhaust generated under urban driving conditions. Following an immediate primary dilution, a minor proportion of the exhaust (< 10%) was fed into the HEPA (High efficiency particulate air) filtered atmosphere in a ventilation shaft leading to the chamber, thereby providing a secondary dilution. During filtered air exposures, the exhaust connection was closed and all the exhaust was shunted away. A schematic figure of the exposure setup can be found in Additional file 2.

During the BD100 exhaust exposures, we used the same detailed tuning that resulted in a steady state atmosphere in the exposure chamber of around 300 μg/m^3^ PM_10_, when using a standard petrodiesel fuel in the engine. This means that the same load and engine rounds per minute (rpm) pattern were used for BD100 exhaust exposures, as would give the petrodiesel fuel exhaust atmosphere previously studied. No modifications of the diesel engine, exhaust dilution system or exposure chamber were done. The reason for this was to study the health effects of the exhaust from a specific vehicle when replacing petrodiesel fuel with BD100 across the same driving conditions. The exposure conditions were similar as in a companion study conducted during the same session, but focused on investigating cardiovascular effects of BD100 exhaust exposure in a separate group of subjects, as reported elsewhere [30].

On-line equipment continuously measured nitric oxides (NO_x_) by chemiluminescence.

and total gaseous hydrocarbons (THC) by flame ionization detector (FID). Real-time monitoring of PM_10_ were performed during the exposures with a tapered-element oscillating microbalance (TEOM, Rupprecht & Patashnik, Albany, New York, USA), to achieve steady state petrodiesel PM levels aiming at 300 μg/m^3^. The fuel was then switched to BD100 with all settings maintained, including the same engine load and speed. After steady state conditions were achieved, i.e. at least 30 min running with the BD100, the subjects were allowed to enter the exposure chamber for the exposure.

PM_10_ concentration was determined gravimetrically using PTFE filters (Pall Teflo Life Science 47 mm, 2 µm) prior to the subjects entering the chamber. Additional filters were collected during the exposures for exposure quality control. Particle number concentration and size distribution (18–638 nm) were determined using a scanning mobility particle sizer system (SMPS), which included an electrostatic classifier platform (TSI 3080, TSI GmbH, Minnesota, USA) with a Differential Mobility Analyser (TSI, DMA 3081) and an ultrafine Condensation Particle Counter (TSI, CPC 3025A), as referred to in our preceding companion paper by Unosson et al*.* [30]. The particle number concentration for BD100 was 2.2 ± 1.4 × 10^5^/cm^3^, particle bound PAHs 76 ± 24 ng/m^3^, semivolatile PAHs 225 ± 167 ng/m^3^ and elemental carbon/organic carbon-ratio 0.43 ± 0.03.

The particle size and number distribution shifted from a mono-modal distribution (peak at 100–140 nm) for petrodiesel, to a bi-modal distribution of higher numbers of smaller particles (one peak at 80–90 nm and a second peak at 30–40 nm) for the BD100 exhaust.

Mass size-fractionated PM was sampled by a Dekati Gravimetric Impactor (DGI, Dekati Ltd, Tampere, Finland) for subsequent chemical and toxicological evaluation. The DGI classifies particle size according to aerodynamic diameter (cut-points; 0.2, 0.5, 1.0 and 2.5 μm) using 47 mm PTFE plates, together with a 70 mm PTFE filter as back-up filters for collection of the < 0.2 μm fraction. A thermal-optical carbon analyzer (Sunset Laboratory Inc, Portland, Oregon, USA) was used to analyse the content of organic carbon (OC) and elemental carbon (EC) applying the EUSAAR 2 thermal protocol. The physical and chemical characterisation of the exposures were as previously described in a companion manuscript [30].

### Bronchoscopy sampling

Bronchoscopy with endobronchial sampling of bronchial mucosal biopsies, bronchial wash (BW) and bronchoalveolar lavage (BAL) was performed six hours after the exposures ended, using an Olympus BF IT200 videobronchoscope during topical anaesthesia according to principles previously described [16]. The bronchoscopy sampling was randomised according to a crossover system, which allowed for biopsies and lavages being sampled contra-laterally at each bronchoscopy, without sampling at the same locations twice.

Bronchial mucosal biopsies were collected from secondary and tertiary carinas using flexible fenestrated forceps and were fixed in acetone containing protease inhibitors, embedded in glycolmetachrylate (GMA) and stored at −20°C until immunohistochemical analysis. BW was performed in the left lung lingula lobe/right middle lobe by introducing 20 ml of saline through the instrument channel, with the bronchoscope tip wedged in a segmental bronchus. Gentle suctioning collected the fluid. BAL was subsequently performed using 3 × 60 ml of saline, which was gently suctioned back after each aliquot. The recovered fluid was filtered through a nylon filter and centrifuged at 400 *xg* for 15 min. Cell pellets were resuspendend in PBS to achieve a cell concentration of 10^6^ cells/ml and the supernatants were frozen at − 80 °C until further analyses. Differential cell counts were performed on slides made by cyto-centrifuge preparation and stained with May-Grünwald Giemsa. Five hundred cells per slide were counted.

### Immunohistochemistry

The immunostaining procedure has been described previously [9, 16, 54]. In short, GMA embedded biopsies were sectioned at 2 µm and collected on glass slides for the immunohistochemical staining. The sections were treated with sodium azide and hydrogen peroxide solution to inhibit endogenous peroxidases. Nonspecific antibody binding was blocked by undiluted culture medium (Sigma; St Louis, Missouri). Primary antibodies were added and incubated overnight, including antibodies for detection of neutrophils, mast cells, macrophages (CD68), CD8 (DAKO, Glostrup, Denmark), eosinophils (Diagnostic Development, Uppsala, Sweden), CD4 (BioLegend, San Diego, CA, USA), EN4, P-selectin (Serotec, Oxford, UK), ICAM-1 (Invitrogen,Carlsbad, CA, USA). Biotinylated rabbit anti-mouse secondary antibodies (IgG F[ab’]_2_; Dako) were applied and incubated for 2 h, followed by streptavidin–biotin horseradish peroxidase complex (Vector Laboratories, Newark, CA, US) for another 2 h. The sections were visualized using 3-amino-9-ethylcarbazole (AEC; Vector Laboratories, Newark, CA, US) and counterstained with Mayer’s haematoxylin. Positively stained nucleated cells were counted within intact epithelium and submucosa, avoiding smooth muscle and glands. Stained cells per submucosal area and epithelial length were calculated using a LeicaQWin V3 system (Leica Q500IW; Leica, Cambridge, UK). Vascular adhesion molecule expression was calculated as the ratio of P-selectin and ICAM-1 positive vessels to the pan-endothelial marker EN4 and presented in percentage.

### Analysis of soluble components

Myeloperoxidase (MPO), Matrix metalloproteinase-9 (MMP-9), Matrix metalloproteinase −12 (MMP-12), Tissue inhibitor of Metalloproteinases 1 (TIMP1), Tissue inhibitor of Metalloproteinases 2 (TIMP2) (R&D systems, Abingdon, UK) and soluble scavenger receptor CD163 (sCD163) (Bender Medsystems GmbH, Vienna, Astria) were analysed using commercial ELISA assays, as per the manufacturer’s instructions.

### Lung function

FEV_1_ and VC were measured by spirometry (Jaeger Masterlab, Carefusion, San Diego, California) according to ATS/ERS guidelines before and after exposures, as well as prior to the bronchoscopy.

### Data analysis and statistics

Wilcoxon signed-rank test was used for comparison of BW, BAL and immunohistochemical data. A p value of 0.05, or less, was considered significant and data are presented as median and interquartile range. Following confirmation of normality by the Shapiro–Wilk test, paired sample T-test was used for lung function data. A p value of 0.05, or less, was considered significant and data are presented as mean with ± SD. Correlations were analyzed using the Spearman rank order correlation test.

All statistical analyses were performed using SPSS version 29 for Macintosh (SPSS Inc., Chicago, USA). Graphical presentations were performed using GraphPad Prism for Macintosh, version 10 (San Diego, CA, USA).

## Supplementary Information

Additional file 1

Additional file 2

## Data Availability

No datasets were generated or analysed during the current study.
