# Supplementary material for: Acute airway inflammation following controlled biodiesel exhaust exposure in healthy subjects
Source: Part Fibre Toxicol. 2024 Dec 5;21:53. doi: 10.1186/s12989-024-00614-5 (PMC11619701; doi:10.1186/s12989-024-00614-5)
Supplement: Supplementary file 1 — Additional file 1 [file 12989_2024_614_MOESM1_ESM.docx]

|  | **Air exposure** | | **BD100 exposure** | | **Air vs BD100 exposure** |
| --- | --- | --- | --- | --- | --- |
| N=14 | Pre exposure | Post exposure | Pre exposure | Post exposure | p-value |
| **VC (L)** | 4.95 ± 0.95 | 4.89 ± 1.01 | 4.88 ± 0.97 | 4.86 ± 1.02 | 0.279 |
| **FEV1 (L)** | 3.95 ± 0.68 | 4.07 ± 0.75 | 3.91 ± 0.63 | 4.03 ± 0.65 | 0.985 |
| **FEV1/VC** | 0.80 ± 0.06 | 0.84 ± 0.07 | 0.81 ± 0.07 | 0.84 ± 0.07 | 0.683 |

**Additional file 1**. Lung function

Lung function data are presented as mean ± standard deviation for air exposure and biodiesel exhaust exposure. Paired Samples T-test was used to compare the change in lung function after air exposure with the change in lung function after biodiesel exhaust exposure. Subjects acted as their own control. Statistical analyses were performed in SPSS.

VC = vital capacity, FEV1 = forced expiratory flow in first second, FEV1/VC is ratio of these parameters and used as an indicator of airway obstruction.
