# Supplementary figures and images for: Acute airway inflammation following controlled biodiesel exhaust exposure in healthy subjects
Source: Part Fibre Toxicol. 2024 Dec 5;21:53. doi: 10.1186/s12989-024-00614-5 (PMC11619701; doi:10.1186/s12989-024-00614-5)

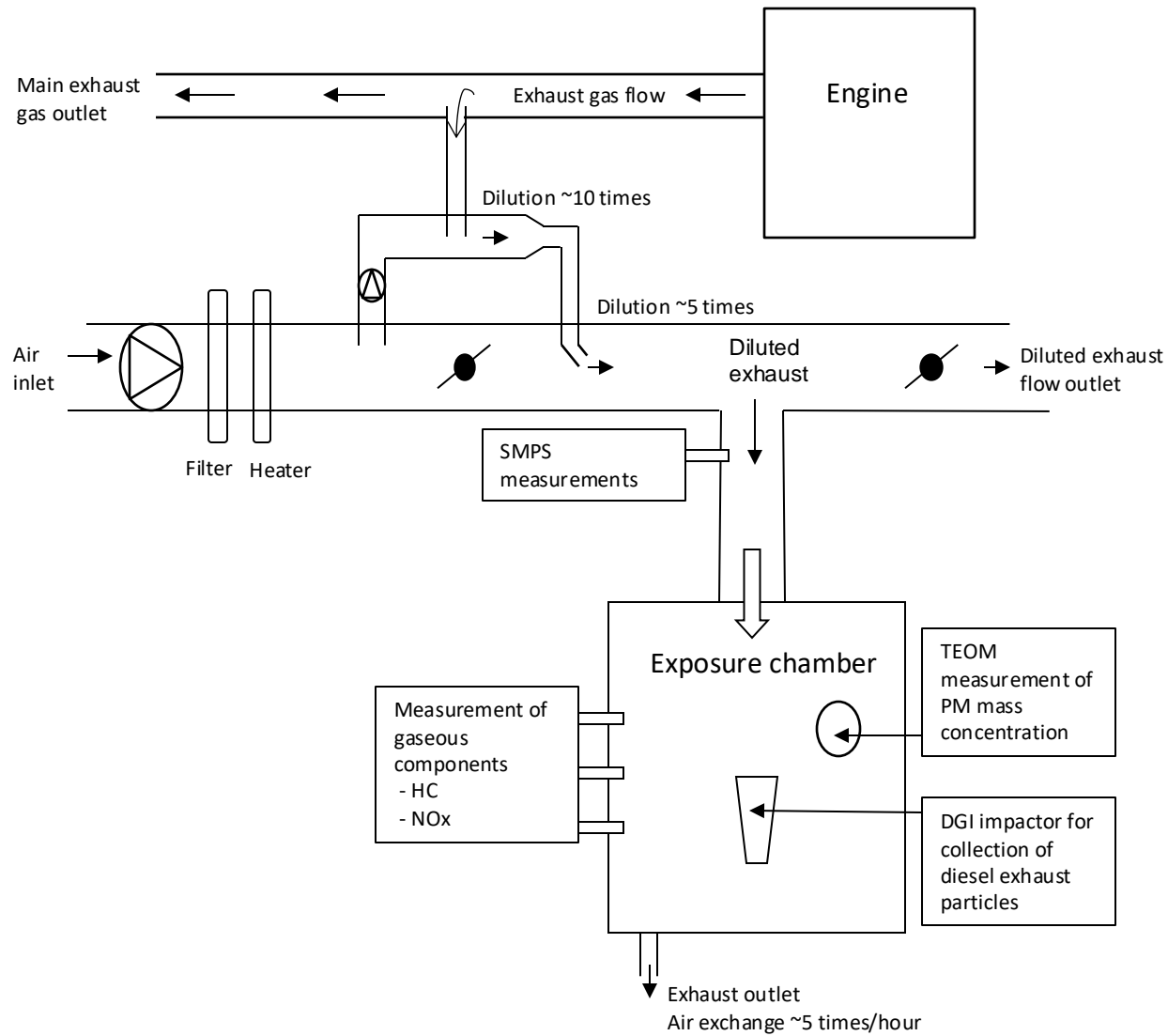

Supplement: Supplementary file 2 — Additional file 2 [file 12989_2024_614_MOESM2_ESM.pdf]
